# Profiling of gut microbial dysbiosis in adults with myeloid leukemia

**DOI:** 10.1002/2211-5463.13193

**Published:** 2021-06-24

**Authors:** Dandan Yu, Xiaomin Yu, Aifang Ye, Chunquan Xu, Xiaolong Li, Wujun Geng, Liqing Zhu

**Affiliations:** ^1^ Department of Clinical Laboratory Sciences The First Affiliated Hospital of Wenzhou Medical University China; ^2^ Department of Translational Medicine Center The First Affiliated Hospital of Wenzhou Medical University China; ^3^ Department of Anesthesiology The First Affiliated Hospital of Wenzhou Medical University China

**Keywords:** gut microbiota, myeloid leukemia, gene sequencing, pathogenesis

## Abstract

Dysregulation of gut microbiota is implicated in the pathogenesis of various diseases, including metabolic diseases, inflammatory diseases, and cancer. To date, the link between gut microbiota and myeloid leukemia (ML) remains largely unelucidated. Herein, a total of 29 patients with acute myeloid leukemia (AML), 17 patients with chronic myeloid leukemia (CML), and 33 healthy subjects were enrolled, and gut microbiota were profiled via Illumina sequencing of the 16S rRNA. We evaluated the correlation between ML and gut microbiota. The microbial α‐diversity and β‐diversity exhibited significant differences between ML patients and healthy controls (HCs). Compared to healthy subjects, we found that at the phylum level, the relative abundance of *Actinobacteria*, *Acidobacteria*, and *Chloroflexi* was increased, while that of *Tenericutes* was decreased. Correspondingly, at the genus level in ML, *Streptococcus* were increased, especially in AML patients, while *Megamonas* (P = 0.02), *Lachnospiraceae* NC2004 group, and *Prevotella 9* (P = 0.007) were decreased. Moreover, ML‐enriched species, including *Sphingomonas*, *Lysobacyer*, *Helicobacter*, *Lactobacillus*, *Enterococcus,* and *Clostridium* *sensu stricto* 1, were identified. Our results indicate that the gut microbiota was altered in ML patients compared to that of healthy subjects, which could contribute to the elucidation of microbiota‐related pathogenesis of ML, and the development of novel therapeutic strategies in the treatment of ML.

## Graphical abstract

The gut microbiota was altered in myeloid leukemia (ML). At the phylum level, the abundance of *Actinobacteria*, *Acidobacteria*, and *Chloroflexi* increased, while that of *Tenericutes* decreased compared to healthy subjects. At the genus level in ML, *Streptococcus* increased while *Megamonas, Lachnospiraceae* NC2004 group, and *Prevotella 9* decreased. ML‐enriched species included *Sphingomonas*, *Lysobacyer*, *Helicobacter*, *Lactobacillus*, *Enterococcus,* and *Clostridium* *sensu stricto* 1.

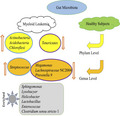

**Abbreviations**
AML: acute myeloid leukemia
ASVs: amplicon sequence variants
CML: chronic myeloid leukemia
FMT: fecal microbiota transplantation
HC: healthy control
MICM: cytogenetic and molecular
ML: myeloid leukemia
MM: multiple myeloma
PCA: principal component analysis
PCoA: principal coordinate analysis
PD: phylogenetic diversity
RF: random forest
SDI: Shannon diversity index

Myeloid leukemia (ML) is a type of hematological malignancy affecting myeloid tissue characterized by dysregulated proliferation and differentiation of myeloid cells, including acute myeloid leukemia (AML) and chronic myeloid leukemia (CML) [1]. Studies have shown that genetic and nongenetic factors such as age, ionizing radiation, and other environmental risk factors were involved in the incidence of ML [2, 3]. However, the profound associations between these factors and ML remain uncertain and there is an urgent need to identify novel biomarkers for clinical diagnosis and therapeutic strategies.

Gut microbiota, one of the largest groups of symbiotic microorganisms in the gastrointestinal tract of human and animals, has been shown to be involved in various diseases such as obesity, diabetes, chronic kidney disease, atherosclerosis, inflammatory bowel diseases, colorectal cancer, endotoxemia, and chronic inflammation [4, 5, 6, 7, 8, 9, 10, 11, 12]. Moreover, studies have revealed the potential association between gut microbiota and acute lymphoblastic leukemia (ALL) [13, 14, 15], while the relationship between gut microbiota and ML remains to be elucidated.

The 16SrRNA gene contains highly conserved sequences in all bacteria that can be targeted by universal PCR primers. It is extremely useful for phylogenetic classification of specific species within the gut microbiota. Herein, we sought to investigate the profile of fecal microbiota in patients with AML, CML, and healthy controls (HCs) and explore its potential implication in ML using 16S rRNA gene sequencing.

## Materials and methods

### Patients

This study was approved (approval number: 2019232) by the Ethics Committee of the First Affiliated Hospital of Wenzhou Medical University, Wenzhou, China, and complied with the 2008 Declaration of Helsinki guidelines. Written informed consent has been obtained from each subject. The study cohort involved 79 participants, including 29 AML patients, 17 CML patients, and 33 healthy subjects. 29 AML patients (19 males, 10 females) with an age range of 14–80 years (median age, 50 ± 20 years) newly diagnosed at the First Affiliated Hospital of Wenzhou Medical University from January 2017 through June 2018 were included in this study. Diagnoses were established according to morphological, immunological, cytogenetic and molecular (MICM) criteria. According to French‐American‐British (FAB) classification, the AML patients were classified as M0 (AML with minimal differentiation), M2 (subtype 2 AML), M3 (acute promyelocytic leukemia), M4 (acute myelomonocytic leukemia), M4Eo (acute myelomonocytic leukemia with eosinophilia), and M5 (acute monocytic leukemia). Fecal samples from all AML patients (M0: 1 patients; M2: 1 patients; M3: 9 patients; M4: 7 patients; M4Eo: 2 patients; M5: 9 patients) were collected before antibiotic treatment and were stored at −80 °C freezer for the subsequent analyses. Antibiotics was used for routine care for fever in our hospital. 17 CML patients in chronic phase (15 males, 2 females) with an age range of 18–66 years (median age 46 ± 13 years) were also recruited from January 2017 through June 2018 at the First Affiliated Hospital of Wenzhou Medical University. 33 age‐matched HCs (17 males, 16 females) with an age range of 21–74 years (median age 46 ± 15 years) were included as controls. Fever within 1 week, medications, pregnancy, and history of any chronic disease were ruled out in all healthy control subjects.

### S rRNA gene sequencing and bioinformatic analysis

The V4 region of the 16S rRNA gene was amplified using dual‐indexed V4‐region primer (515F, 5′‐GTGCCAGCMGCCGCGGTAA‐3′, and 806R 5′‐GGACTACHVGGGTWTCTAAT‐3′; Rohnin Biosciences, China) with barcodes. All polymerase chain reactions (PCRs) were carried out with Applied Biosystems® Gene Amp® PCR System 9700. Illumina Hiseq Rapid SBS Kit V2 (FC‐402‐4023 500 cycles) was used for PE250 sequencing. Library quality was evaluated on the Qubit® 2.0 Fluorometer (Thermo Scientific). The library was sequenced on an Illumina HiSeq2500 platform and 250‐bp paired‐end reads were generated. 16S rRNA gene sequencing was performed at Rohnin Biosciences, China. To generate taxon bins with unique taxonomy, OTUs at 97% identity threshold with the same taxonomic classification were combined into a single bin. α‐ and β‐diversity metrics were calculated using r language in in the analysis of microbiome communities. The Shannon Diversity Index (SDI) was used for α‐diversity calculations, and weighted and unweighted UniFrac for β‐diversity distances [16]. To find out which species are responsible for the differences in β‐diversity, Random Forest (RF) was used to identify the signature associated with the subjects and important species that differ significantly between groups [17, 18]. It is a Decision Tree classifier widely used in the field of biology that can handle a large number of input variables and evaluate the importance of each [18, 19]. Here, it can be used to find biomarkers identifying species in these groups and combined with abundance information allowing for a comprehensive analysis of the Gini index for each characteristic (i.e., each classification level or different taxa). A heat map was created to complement this analysis.

### Statistical analysis

Alpha diversity and principal coordinates were evaluated using the Wilcoxon rank sum test. PERMANOVA was utilized to examine microbial community clustering using weighted, unweighted, and Bray–Curtis distance matrices. Correlations between variables were calculated using Spearman's rank correlation. *P*‐values < 0.05 were considered significant. The data were analyzed using spss version 17.0 (IBM SPSS Stastics 21: IBM Corporation, Armonk, NY, USA).

## Results

### Cohort description and sequencing data

**Fig. 1:**
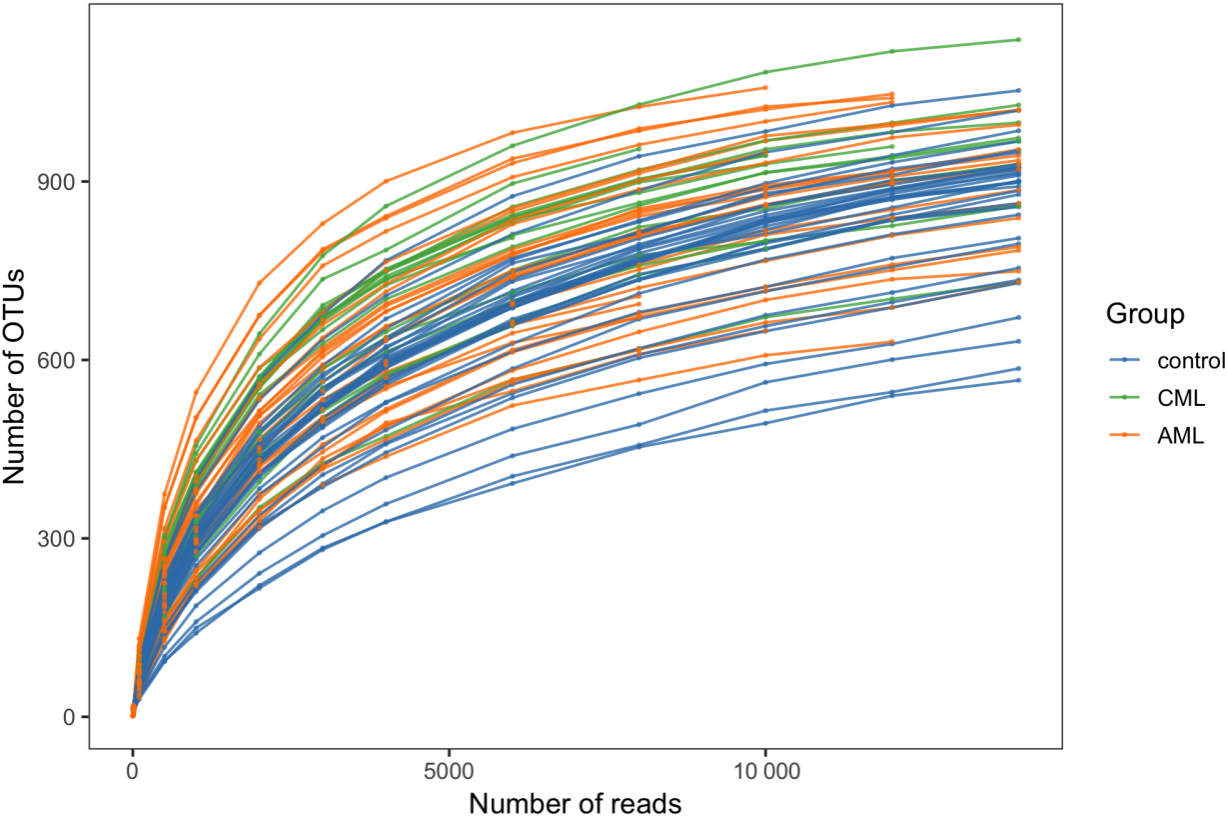
Rarefaction curve. *X*‐coordinate: the number of randomly selected sequencing bars; *Y*‐coordinate: the α‐diversity index calculated based on the number of sequencing bars. The curves of different colors represent different samples.

A total of 79 fecal samples were collected for next‐generation sequencing. The next‐generation sequencing studies provided 2 652 921 valid tags with an average read length of 286.54 base pairs, ranging from 277 to 465 bp. The flattening trend for the rarefaction curve of all the samples reflected the sufficiency of the sequencing quantity (Fig. 1).

### Alpha diversity

**Fig. 2:**
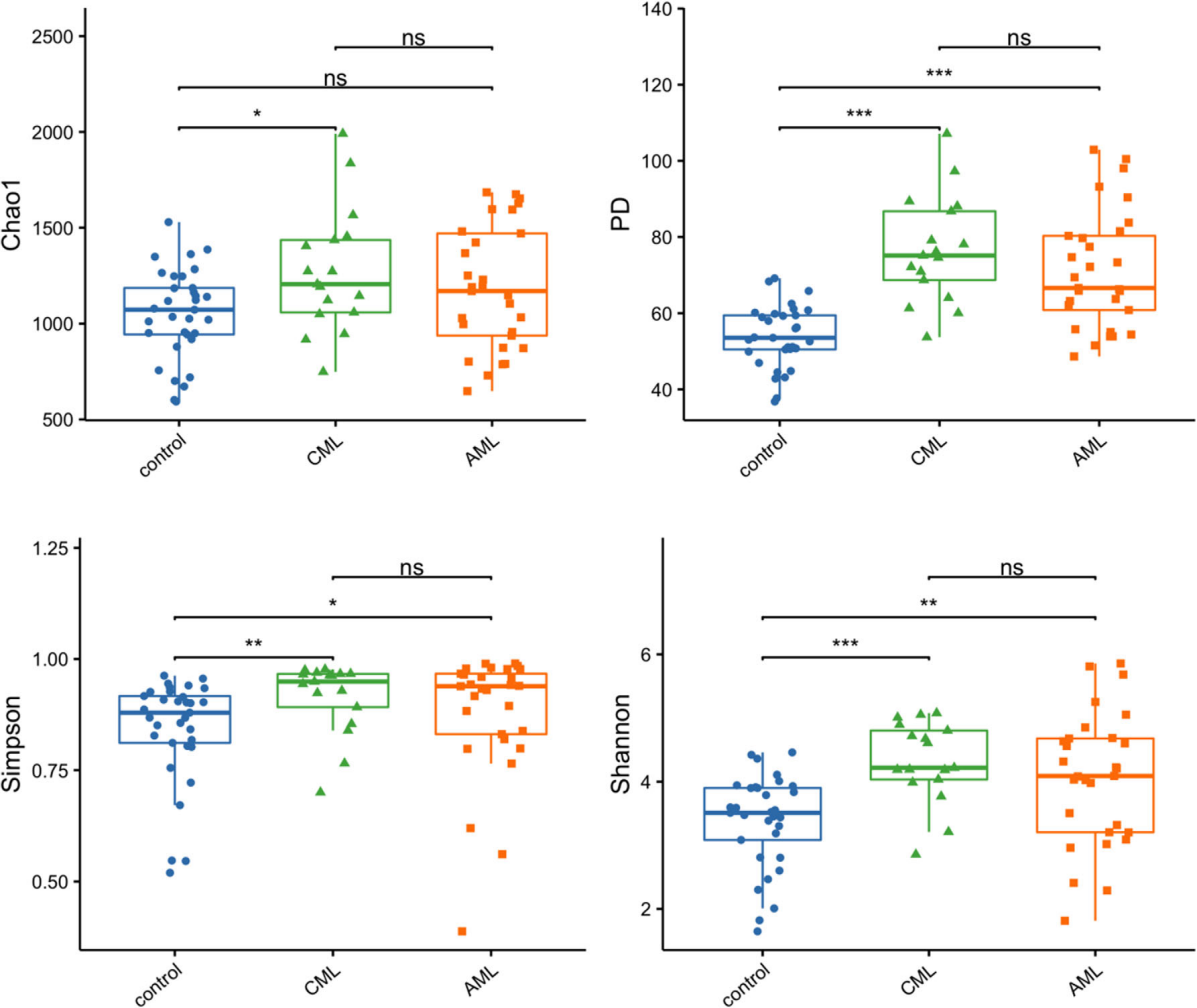
The α‐diversity indices between myeloid patients (*P* group) and HCs (*N* group). The α‐diversity was evaluated using the Wilcoxon rank sum test (*n* = 29 for AML; *n* = 17 for CML; *n* = 33 for HCs). α‐diversity indexes include Chao 1(measurement of total richness), PD whole tree (sum of all branch‐lengths on the constructed phylogenetic tree from all taxa), Simpson (richness and evenness), and Shannon (species numbers and evenness of species abundance). *P < 0.05, **P < 0.01, and ***P < 0.001.

Alpha diversity was used to assess the richness, or the number of species within samples, and their evenness using an independent variable Wilcox test. The OTUs in AML patients, CML patients, and health controls are 799.62 ± 215.79, 871.47 ± 180.07, and 608.12 ± 133.36, respectively. In Fig. 2, significant differences were shown in the phylogenetic diversity (PD) whole tree, Simpson and Shannon index between ML group and control group, while there was no significant difference between CML and AML. There was a dramatic difference in Chao 1 between control and CML groups, while no significant differences were found between other groups. The result of high bacterial diversity in ML patients confirmed by the alpha diversity was consistent with the findings in patients diagnosed with colorectal malignancies or liver cancer [20, 21].

### Beta diversity

**Fig. 3:**
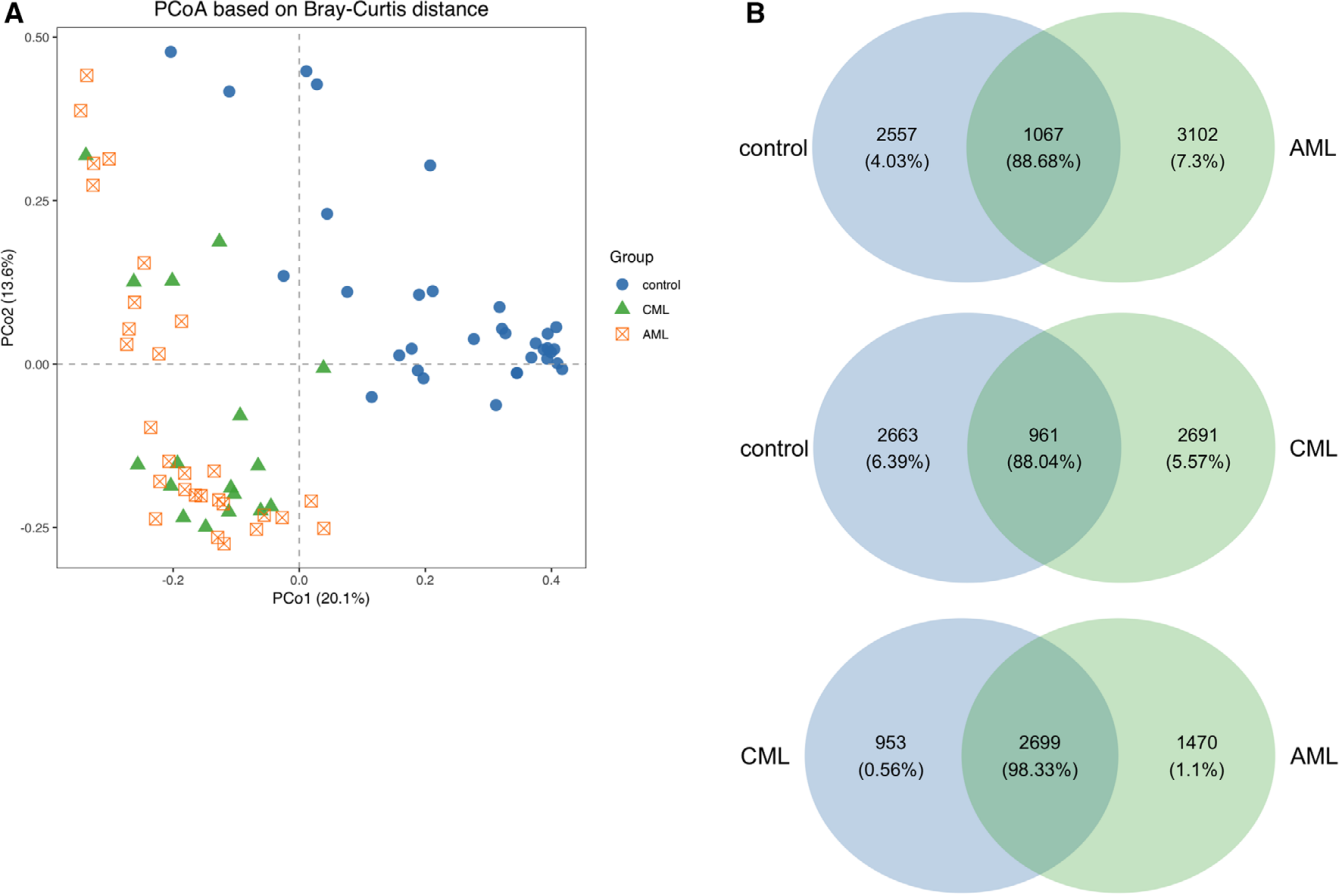
The beta‐diversity and Venn diagram (based on genera) between myeloid patients (*P* group) and HCs (*N* group). (A) PCA. Each dot represents a sample. The plot shows a distinct difference between ML patients and HCs. (B) Venn plot. The AML and control groups have 1607 shared genera, with 3102 unique genera in the AML group and 2557 unique genera in the control group. The two groups between the CML and control group have 961 shared genera, with 2691 unique genera in the CML group and 2663 unique genera in the control group. The two groups between the AML and CML group have 2699 shared genera, with 1470 unique genera in the AML group and 953 unique genera in the CML group.

Beta diversities for comparing microbial composition between the groups were determined based on the differences in shared species and their abundance. Gut microbiota from patients with AML, CML, and HCs were distinguished by principal component analysis (PCA), principal coordinate analysis (PcoA), and NMDS analysis. The Vegan package was used for PCA and NMDS analysis, and APE package was used for PcoA [22]. All results indicated that the beta diversity of the gut microbiota was altered by AML and CML. According to the PCA, PCoA, and NMDS analysis, the AML and HC, as well as the CML and HC, were separated into distinct clusters. These distinct clusters were not observed in the AML and CML (Fig. 3).

### Relative abundances of bacterial taxa at different taxonomic levels in AML, CML, and control group

**Fig. 4:**
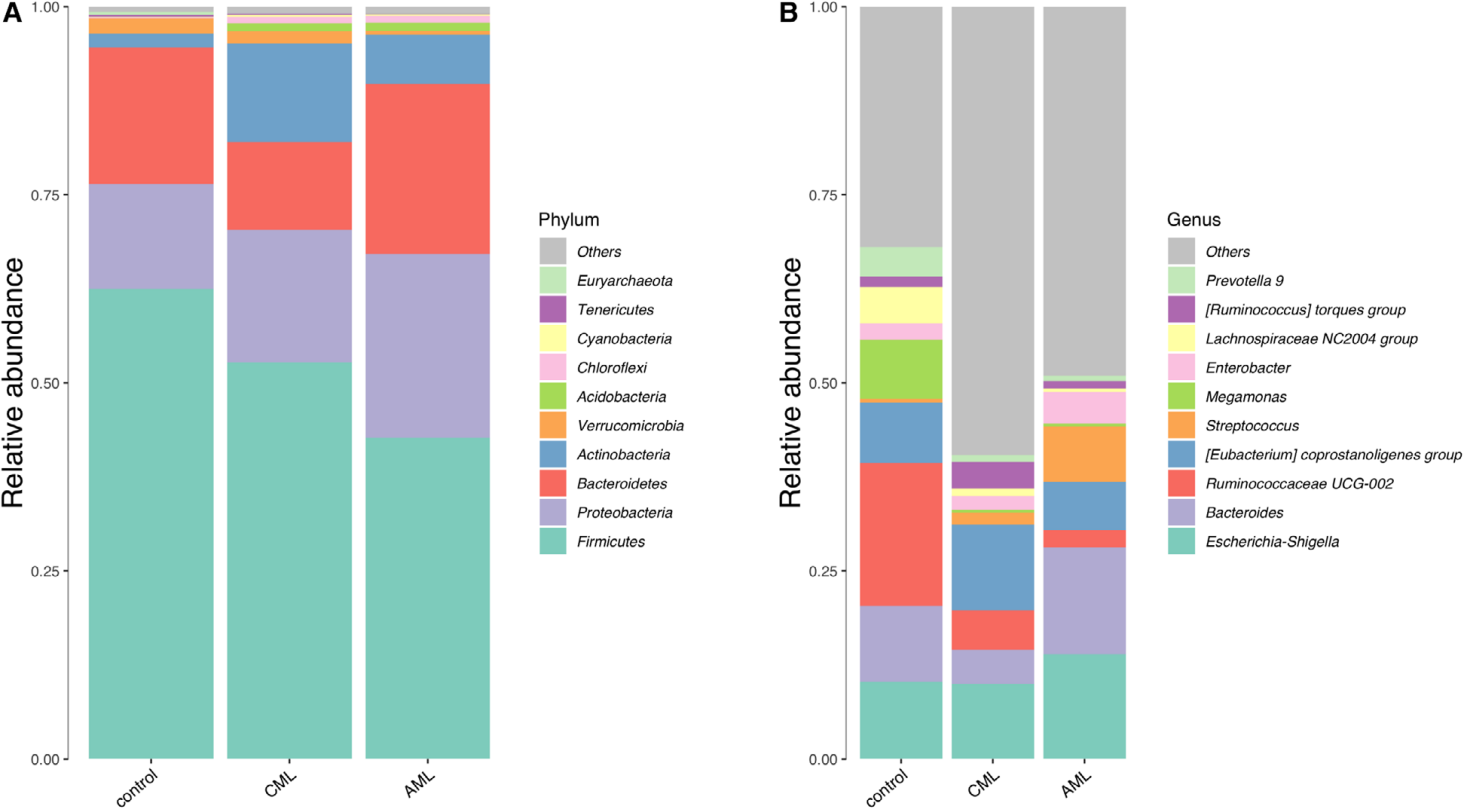
Stacked bar graphs for the relative abundance of bacterial 16S rRNA from fecal samples of CML, AML, and control group at the phylum and the Genus levels. (A) The relative abundance of bacterial microbiota at the phylum level and (B) genus level.

As shown in Fig. 4a,b, compared to the heathy controls, the top 10 OTUs were significantly different in AML patients, which included higher relative abundances of phylum *Actinobacteria*, *Acidobacteria,* and *Chloroflexi*, and lower relative abundances of phylum *Firmicutes* and *Tenericutes*. The top 10 OTUs were significantly different in CML patients compared to the heathy controls as well, demonstrating higher relative abundances of phylum *Actinobacteira*, *acidobacteria*, and *Chloroflexi*, with lower relative abundances of phylum *Tenericutes* (Fig. 4A and Table 1).

**Table 1:** Differences in microbiota between ML patients (*P* group) and HCs (*N* group) in phylum level.

Phylum	Mean_control	Mean_CML	Mean_AML	SD_control	SD_CML	SD_AML	*P* value1	*P* value2
*Firmicutes*	0.624767518	0.527259599	0.426865402	0.165096495	0.161396124	0.187348242	0.052	0.000
*Proteobacteria*	0.139915422	0.176218217	0.244494306	0.192545545	0.147613844	0.247129115	0.500	0.066
*Bacteroidetes*	0.18131259	0.116742729	0.225997477	0.12208926	0.112362377	0.19635138	0.075	0.295
*Actinobacteria*	0.018244656	0.131247064	0.065686142	0.037963922	0.147995158	0.08285978	0.007	0.007
*Verrucomicrobia*	0.020050343	0.015899388	0.00470537	0.077105848	0.034129971	0.005184236	0.834	0.263
*Acidobacteria*	7.58388E‐05	0.010417324	0.010697011	8.8474E‐05	0.002418282	0.008453029	0.000	0.000
*Chloroflexi*	0.000993128	0.008706808	0.009012119	0.000605971	0.003109845	0.009206873	0.000	0.000
*Cyanobacteria*	0.000928123	0.002551754	0.002124608	0.003038058	0.002719593	0.003775387	0.070	0.172
*Tenericutes*	0.002968549	0.001065567	0.000673957	0.004298617	0.001476807	0.000589265	0.027	0.005
*Euryarchaeota*	0.003777496	0.000322474	0.000526015	0.012387694	0.000328448	0.001816765	0.119	0.146

*P* value1: Control group vs CML; *P* value2: Control group vs AML.

We then explored the differences of the relative abundances of the gut microbiome between ML patients and controls at the genus level (Fig. 3B). Compared to HCs, the top 10 OTUs in AML patients had significantly higher relative abundances of genus *Streptococcus*, while the relative abundance of genus *Ruminococcaceae* UCG‐002, *Megamonas*, *Lachnospiraceae* NC2004 group, and *Prevotella* 9 was decreased. Compared to the HCs, the top 10 bacteria in CML patients had significantly higher relative abundances of genus *Streptococcus* and *Ruminococcus torques* group, while decreases in the relative abundance of genus *Bacteroides, Ruminococcaceae* UCG‐002, *Megamonas*, *Lachnospiraceae* NC2004 group, and *Prevotella* 9 were observed (Fig. 4B and Table 2).

**Table 2:** Differences in microbiota between ML patients (*P* group) and HCs (*N* group) in phylum level. *P* value1: Control vs CML. *P* value2: Control vs AML. *P* value3: CML vs AML.

Genus	Mean_control	SD_control	Mean_CML	SD_CML	Mean_AML	SD_AML	*P* value1	*P* value2	*P* value3
*Escherichia‐Shigella*	0.10274356	0.17415626	0.09970066	0.131408334	0.13950497	0.204720184	0.95	0.448	0.477
*Bacteroides*	0.101129276	0.099197306	0.045756308	0.081022201	0.141633688	0.156791393	0.041	0.238	0.009
*Ruminococcaceae UCG‐002*	0.189774036	0.132228098	0.052184764	0.049861158	0.023304937	0.021965665	0	0	0.035
*[Eubacterium] coprostanoligenes group*	0.080324157	0.079047273	0.113952624	0.101948896	0.064030016	0.070176642	0.203	0.397	0.086
*Streptococcus*	0.004676728	0.009769969	0.015857326	0.021373347	0.073785953	0.114815681	0.014	0.003	0.013
*Megamonas*	0.078749598	0.175002993	0.00358928	0.002864822	0.003825938	0.006941338	0.019	0.02	0.894
*Enterobacter*	0.021895754	0.034570794	0.018486193	0.016950403	0.041616839	0.116825531	0.704	0.358	0.424
*Lachnospiraceae NC2004 group*	0.048052928	0.074438347	0.009821447	0.013170695	0.00470126	0.004599962	0.007	0.002	0.139
*[Ruminococcus] torques group*	0.013925454	0.013364951	0.035493211	0.02506085	0.009986069	0.008481887	0.003	0.178	0.001
*Prevotella 9*	0.039	0.064	0.009	0.017	0.007	0.007	0.016	0.007	0.550

### Differences of the gut microbial taxa between ML patients and HCs

**Fig. 5:**
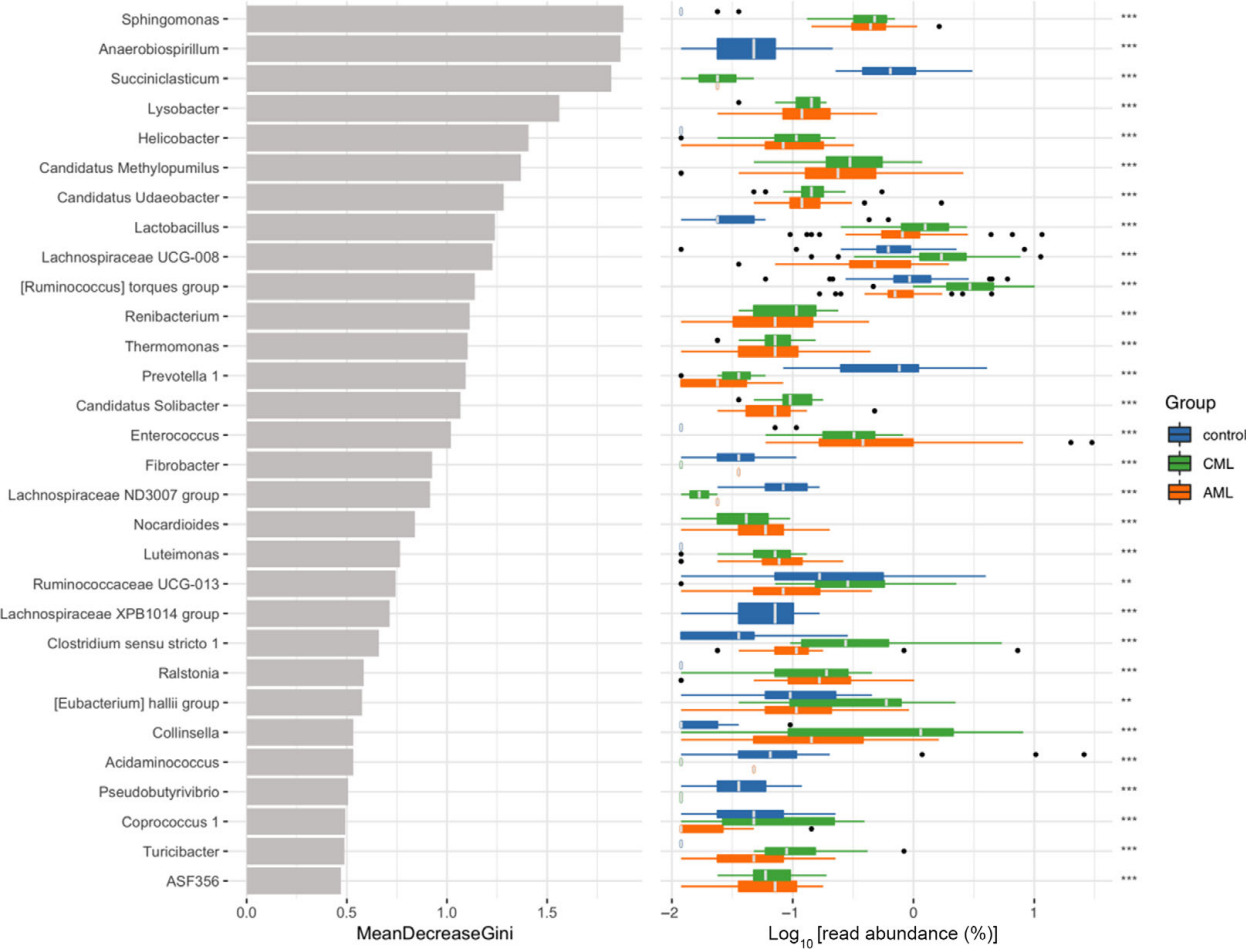
Gut microbial composition of myeloid patients (*P* group) and HCs (*N* group). Left panel: the average decrease value of the Gini index (the horizontal coordinate) and the classification information of genus (the vertical coordinate); right panel: the box diagram of the class group abundance of different groups in which the * represents the significance of differences between groups (Kruskal–Wallis rank sum test; ***P < 0.001; **P < 0.01). Categories that do not have accurate classification information are not analyzed.

We identified ML‐enriched species including *Sphingomonas*, *Lysobacyer*, *Helicobacter*, *Lactobacillus*, *Enterococcus,* and *Clostridium* *sensu stricto* 1, while *Anaerobiospirillum* and *Prevotella* were decreased (Fig. 5, Table 3, and Fig. S1A,B).

**Table 3:** Gut microbial composition.

Predictors	Mean decrease gini	*P* value	Mean_AML	Mean_CML	Mean_control	SD_AML	SD_CML	SD_control
*Sphingomonas*	1.881192594	2.78E‐13	0.492728252	0.44936101	0.006861609	0.303542569	0.173458662	0.008953183
*Anaerobiospirillum*	1.866324865	3.74E‐16	0	0	0.053809457	0	0	0.038400673
*Lysobacter*	1.560073529	4.42E‐14	0.156982646	0.137402119	0	0.104724661	0.045403611	0
*Helicobacter*	1.406932026	6.03E‐14	0.127805243	0.11496912	0.000361137	0.094640532	0.069227055	0.002074576
*Lactobacillus*	1.239296585	8.00E‐13	1.474486211	1.392949028	0.055254006	2.366573957	0.761206121	0.124487514
*Prevotella 1*	1.09253235	1.58E‐13	0.016437973	0.012618562	0.855895386	0.023278177	0.019973239	0.789999788
*Enterococcus*	1.019132146	3.60E‐13	2.644048015	0.335092922	0.008667295	6.589190388	0.225564352	0.02194912
*Clostridium sensu stricto* 1	0.660375079	2.47E‐10	0.369854401	0.825113742	0.033946906	1.339431396	1.399913792	0.054296845

## Discussion

The gut microbiome may exert profound effects on human health. Patients with hematologic malignancy usually exhibit dysbiosis of the intestinal microbiota that can cause damage to the intestinal epithelial barrier, contributing to the progression of leukemia [14, 23]. Thus, the analysis of gut microbiota composition is critical for investigating its association with diseases.

In our study, the gut microbiota of ML patients and control groups were compared using 16S rRNA analysis. We found that *Streptococcus* was the only genus increased in abundance in both the AML and CML microbiomes compared with the control cohort. Interestingly, *Streptococcus* was shown to be enriched in patients with ulcerative colitis and multiple myeloma (MM), proposing a mechanistic explanation for the interaction between MM‐enriched bacteria and MM progression via recycling urea nitrogen [24, 25]. More studies are needed to explore the role of *Streptococcus* in ML progression.

Diversity analyses indicated that the three groups showed different compositions of microbiota. We used RF to find ML‐enriched species including *Sphingomonas*, *Lysobacter*, *Helicobacter*, *Lactobacillus*, *Enterococcus*, and *Clostridium* *sensu stricto 1*, while *Anaerobiospirillum* and *Prevotella* were decreased. Among these species, *Lysobacter* species, a group of environmental bacteria characterized by inherent resistance to numerous antibiotics, has shown its potential as a new supply of antibiotics [26]. *Helicobacter pylori* has been classified as a carcinogen since 1994, although gastric cancer appears in the gastric mucosa epithelium, several observations suggest that the cancer cells may originate from circulating bone marrow stem cells recruited to the mucosa to replace normal gastric stem cells that have underwent apoptosis due to *H. pylori* infection [27]. Gunnar Larfors *et al*. [28] showed that *H. pylori* infection could serve as a risk factor for CML following gastric conditions indicating *H. pylori* infection.

Matthwe S. L. Lee *et al*. [29] reported that a patient with AML and prolonged severe neutropenia was successfully treated with emergent fecal microbiota transplantation (FMT) due to *Clostridioides* *difficile* infection. Kazuhiko Kakihana *et al*. [30] indicated that FMT might shift the systemic allogeneic immune response to an anti‐inflammatory state by changing the intestinal microbiota and might be effective against other forms of acute graft‐versus‐host disease, all of which suggested that FMT might be a promising and safe therapy. Modulating the microbiota by using probiotics or next‐generation beneficial microbes constitutes a future perspective for the development of either nutritional or pharmaceutical tools to maintain health. In this study, we demonstrated that there were differences in intestinal flora between leukemia and healthy control group. Therefore, it remains to be further studied whether increasing the dominant bacteria in healthy control group or reducing the dominant bacteria in leukemia patients can alleviate the progression of leukemia by starting with the bacteria belonging to different bacteria genus.

There were several limitations to our study. First, it is very conservative that only a 97% OTU identity was used in this study, but not amplicon sequence variants (ASVs) or 99% OTU identity. Second, AML comprises a heterogeneous group of neoplastic diseases, in which ≥20% of cells in bone marrow or in the blood are myeloblasts. AML is classified into 6 categories [31], although we did not consider these categories when comparing ML and control groups. Finally, in addition to predictive analysis using PICRUSt, metagenomics and other studies are needed for further collection of more accurate data of the bacteria in patients and to confirm that AML and dysbiosis are significantly related.

## Conclusions

In patients with ML, the relative abundance of *Actinobacteira acidobacteria* and *Chloroflexi* at the phylum level, and *Streptococcus* at the genus level were increased along with the decrease in the relative abundance of *Tenericutes* at the phylum level and *Megamonas, Lachnospiraceae NC2004 group, and Prevotella 9* at the genus level. *Sphingomonas*, *Lysobacyer*, *Helicobacter*, *Lactobacillus*, *Enterococcus,* and *Clostridium* *sensu stricto* 1 were identified as ML‐enriched species. Our findings may be helpful to elucidate the microbiota‐related pathogenesis of ML and contribute to the development of new potential therapeutic strategies.

## Conflicts of interest

The authors declare no conflict of interest.

## Author contributions

DY and LZ conceived and designed the project; DY, AY, XL, and CX acquired the data; DY and WG analyzed and interpreted the data; and DY and XY wrote the paper.

## Supporting information

Fig S1. Heatmaps of the gut microbial taxa between ML patients and HCs. (A) Heatmap‐phylum: compared to the heathy controls, the top 40 bacteria were significantly different in AML patients, which included higher abundances of phylum *Actinobacteria*, *Acidobacteria*, and *Chloroflexi*, and lower relative abundances of phylum *Tenericutes*. (B) Heatmap‐genus: compared to the HCs, the top 50 bacteria in ML patients had significantly higher relative abundances of genus *Streptococcus*. While the relative abundance of genus *Ruminococcaceae* UCG‐002, *Megamonas*, and *Prevotella* 9 were decreased.

## Data Availability

The data that support the findings of this study are available from the corresponding authors (wzzhuliqing@126.com; gengwujun@wzhospital.cn) upon reasonable request.
