# Supplementary figures and images for: Profiling of gut microbial dysbiosis in adults with myeloid leukemia
Source: FEBS Open Bio. 2021 Jun 24;11(7):2050–9. doi: 10.1002/2211-5463.13193 (PMC8406483; doi:10.1002/2211-5463.13193)

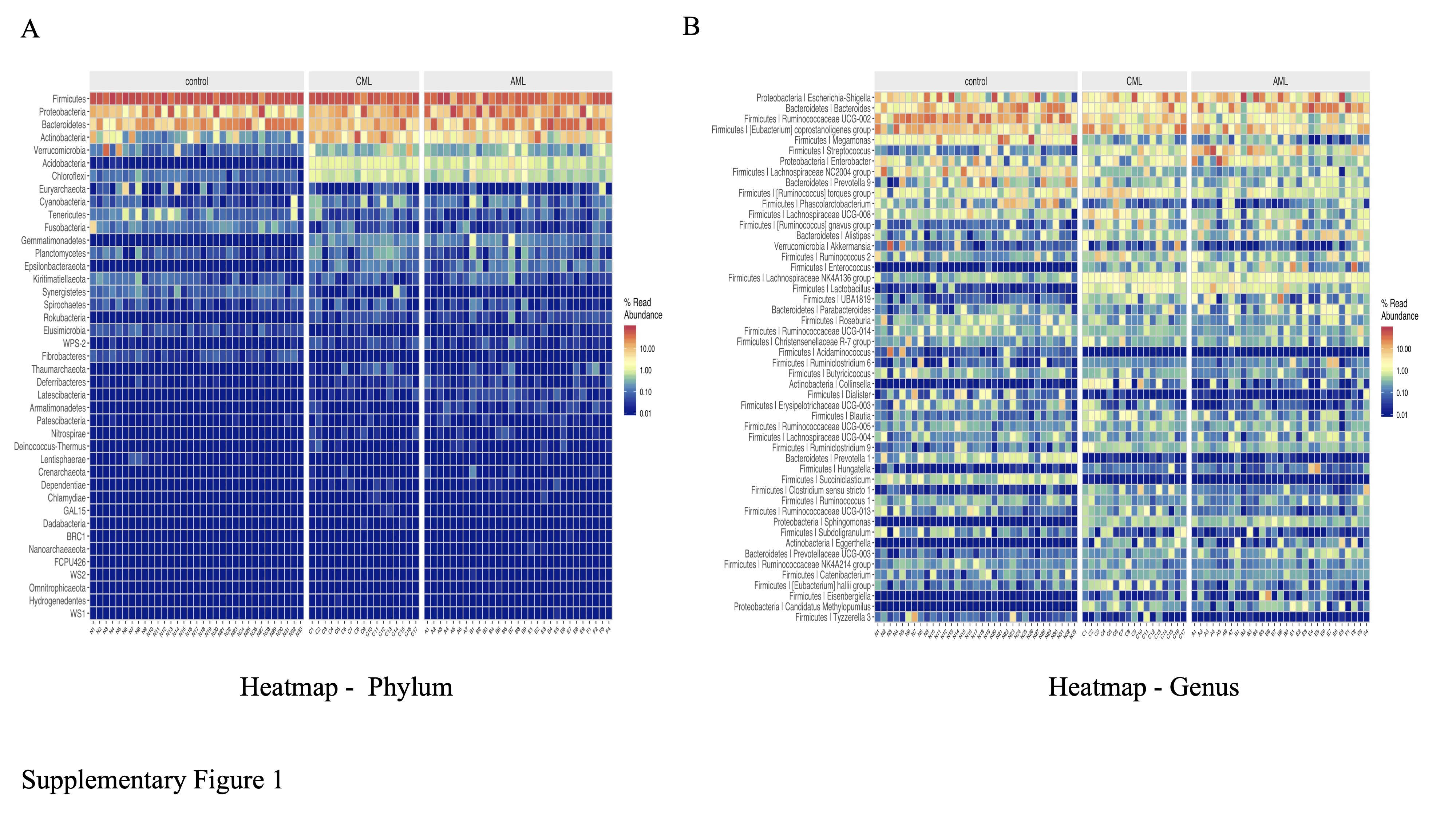

Supplement: Supplementary file 1 — Fig S1. Heatmaps of the gut microbial taxa between ML patients and HCs. (A) Heatmap‐phylum: compared to the heathy controls, the top 40 bacteria were significantly different in AML patients, which included higher abundances of phylum Actinobacteria, Acidobacteria, and Chloroflexi, and lower relative abundances of phylum Tenericutes. (B) Heatmap‐genus: compared to the HCs, the top 50 bacteria in ML patients had significantly higher relative abundances of genus Streptococcus. While the relative abundance of genus Ruminococcaceae UCG‐002, Megamonas, and Prevotella 9 were decreased. [file FEB4-11-2050-s001.jpg]
